# Meaning in life and adolescent self-control: Effect of perceived social support and its gender differences

**DOI:** 10.3389/fpsyg.2022.1087668

**Published:** 2022-12-23

**Authors:** Yafei Liu, Siyu Di, Yaohui Shi, Chao Ma

**Affiliations:** ^1^Normal College, Shihezi University, Shihezi, China; ^2^Center of Application of Psychological Research, Shihezi University, Shihezi, China

**Keywords:** meaning in life, self-control, perceived social support, gender differences, adolescents

## Abstract

**Purpose:**

The objective of this study is to investigate the relationship between meaning in life and adolescent self-control, as well as the role of perceived social support and gender in this pathway.

**Methods:**

For this purpose, a total of 936 adolescents from two high schools were selected as subjects in this study. The Meaning in Life Scale, the Self-Control Scale, and the Perceived Social Support Scale were used for the research.

**Results:**

The results obtained in this study have shown that meaning in life is a significant positive predictor of adolescent self-control and perceived social support. In addition, perceived social support partially mediates the relationship between meaning in life and self-control. Further, it has been found that gender moderates the second half of the pathway of the mediation model of meaning in life on self-control, specifically, perceived social support is a stronger predictor of self-control for females than for males.

**Conclusion:**

The results of this study suggest that enhancing perceived social support promotes self-control in adolescents with lower meaning in life, and this process is more pronounced in females.

## 1. Introduction

Adolescence is an important period in the development of an individual’s life. However, adolescents are prone to show greater paranoia, emotional excitement and impulsive behavior during this period due to the various pressures, conflicts and contradictions they are often faced with. Therefore, good self-control is a necessary condition and basic guarantee for the development of good personality and behavior in adolescents (Wang Hongjiao, 2004). Self-control is an individual’s ability to control impulses to resist temptation and pursue worthy goals (Touré-Tillery and Fishbach, 2015). The lower level of self-control predisposes adolescents to suicidal tendencies, anxiety and depression, as well as increases the likelihood of problematic behaviors such as school bullying and cyber violence (Wenzel et al., 2021). Recent investigations have found that adolescents are frequently exposed to school bullying and cyber violence, which has led to an increase in suicidal ideation and self-harming behaviors, and that those with low self-control are more likely to become repeat victims and perpetrators (Nofziger and Callanan, 2016; Oliva et al., 2019; Hu Qian et al., 2022). Therefore, exploring the formation mechanism of adolescent self-control is an important reference for improving adolescent self-control and promoting the perfection of adolescent personality and positive as well as healthy growth.

Providing a sense of control over life events is one function of the meaning in life (Zhang Shuyue and Haoqiang, 2010). Meaning in life refers to the degree to which an individual seeks, Perceived and appreciates the meaning of his or her life, as well as his or her awareness of the purpose, mission and value of life (Brassai et al., 2011). Research suggests that the powerful psychological restorative and constructive functions of meaning in life enable individuals with high meaning in life to experience higher levels of well-being, maintain higher degrees of self-control, and experience less health anxiety (Hedlund, 1977; Jong Jae Kim, 2012; Zhang Xiuge and Wenyu, 2019). Several studies have found that meaning in life motivates individuals to regulate their emotions and behavior patterns while positively predicting self-control (Nie Hanying, 2017; Schnell and Krampe, 2020). Hedlund noted that a strong meaning in life makes individuals aware of the value of their own existence and clarifies the direction of their lives, which drives intrinsic motivation to regulate external behaviors to align with life goals (Yek et al., 2017). This in turn enhances one’s ability to control oneself. However, the lack of meaning in life leads to a low level of self-control and instinctive impulses to dominate one’s behavior, which eventually leads to many problematic behaviors such as crime and addiction (Jia, 2016; Bernadette and Tatjana, 2019). Adolescents are in a period of important exploration of the meaning of life, and are in an important stage of developing self-control (Hamama and Hamama-Raz, 2021; Ting, 2021). Therefore, it may be more practical to examine the impact of adolescents’ self-control ability from the perspective of meaning in life.

### 1.1. Perceived social support as a mediator

Perceived social support refers to the emotional experience and satisfaction of being respected, understood and supported by others in society, referring to individuals’ subjective perceptions of their own social support level, their expectations and evaluations of social support, and their beliefs about their ability to receive social support (Barbara et al., 1991; Hanxiao, 2008; Ye Baojuan et al., 2014). It has been found that higher levels of perceived social support can help individuals reduce the effects of negative emotions, enhance their ability to deal with stressful events, and decrease feelings of depression (Qiu Yiwen and Yi, 2021; Sun et al., 2021). According to Stillman’s hypothesis that meaning in life promotes interpersonal relationships,” there is a mutually reinforcing symbiotic relationship between the sense of social belonging and meaning in life, which means that a positive sense of social belonging will promote an individual’s meaning in life, and a higher meaning in life will also promote the establishment of better interpersonal relationships (Stillman et al., 2009). Empirical research has shown that the higher an individual’s sense of meaning in life, the more social support they perceive (ZN, 2019). Additionally, perceived social support is closely related to the development of self-control among adolescents. The self-control resource depletion model suggests that social support helps individuals manage and replenish the resources needed for self-control and restore their self-control in a timely manner (Fitzsimons and Finkel, 2011). Several studies have found that social support positively predicts self-control and further increases well-being (HY Jung, 2008; Zhang et al., 2016). Based on this, this study hypothesized that perceived social support may play a mediating role between meaning in life and self-control.

### 1.2. Gender as a moderator

Social cognition and social role theory suggest that the socialization process leads to gender differences and that people tend to imitate same-gender individuals in order to conform to socially expected gender role norms and acquire certain more gender-specific behaviors (Zhang Shupeng and Caina, 2015; Ding Qian and Zongkui, 2020). Does gender play a role in the process of perceived social support mediating meaning in life and self-control? However, this needs to be explored further. Notably, numerous fields of research have demonstrated that self-control is significantly different by gender, but this result is also subject to controversy. Some researchers have explained why there are gender differences in the area of self-control in three ways: physiological, evolutionary, and sociocultural (Van den Akker et al., 2013). Physiologically, males exhibit higher levels of emotional arousal and activity due to androgen secretion, which induces more instinctive impulsivity and aggression, resulting in relatively weaker self-control. In terms of brain development, females have earlier brain development (especially in the left hemisphere of the brain) than males, and thus females have more mature emotional control and social interaction skills. In addition, sociocultural influences make females’ level of self-control significantly higher than that of male. Meanwhile, Hamama found that females reported higher levels of self-control compared to males (Hamama and Hamama-Raz, 2021) and that the gap was more pronounced with age (Kochanska et al., 2001), which provides empirical evidence for the existence of gender differences in self-control. Nonetheless, Jo and Bouffard’s study concluded that among Korean youth aged 10–14 years, boys and girls have largely similar levels of self-control (Youngoh Jo, 2014).

Therefore, it is necessary to continue to explore the gender differences in self-control in order to clarify the mechanisms of its occurrence and development. In addition, gender differences were found to be significant in adolescents’ perceived social support and self-control, with females’ ability to perceive social support significantly better than males (Zhang et al., 2016; Nakash et al., 2022). In addition, the effect of females’ perceived social support on self-control is more significant than that of males’ perceived social support (Orkibi et al., 2018). This may indicate that the difference in self-control between males and females is closely related to their ability to perceive social support. Based on this, the present study hypothesized that gender plays a moderating role in the influence of meaning in life on adolescent self-control through perceived social support, and that perceived social support has a greater effect on self-control for females than for males.

### 1.3. The current study

In summary, this study aims to construct a pathway of “meaning in life → perceived social support → self-control” to systematically examine the influence of meaning in life on self-control of adolescents, and the mediating role of perceived social support between meaning in life and self-control also testing the moderating role of gender differences in the pathway. We propose the following hypotheses.

*Hypothesis 1*: meaning in life is positively related to adolescent self-control and perceived social support.

*Hypothesis 2*: perceived social support is a mediator in the relationship between meaning in life and adolescent self-control.

*Hypothesis 3*: gender differences play a moderating role in the second half of the pathway by which meaning in life affects adolescent self-control through perceived social support.

**Figure 1:**
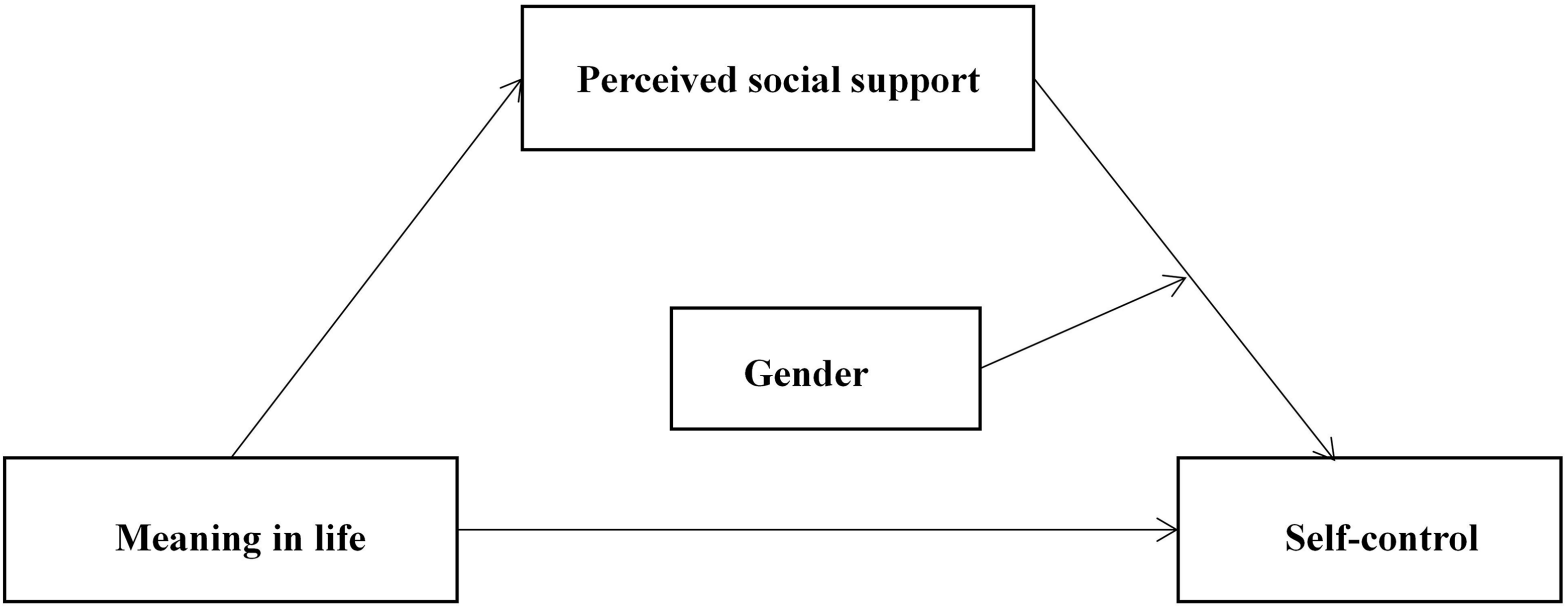
Concept framework.

The proposed model is illustrated in Figure 1.

## 2. Materials and methods

### 2.1. Participants

A stratified whole-group sampling method was used to select 1,006 high school students from two middle schools in China. After obtaining informed consent from the school leaders, adolescents and their parents, the test was administered to the group in a classroom setting. Before the test, the test leader read out the instructions and promised that the contents of the questionnaire would be kept strictly confidential and used for scientific research only, emphasizing the need for subjects to answer truthfully and independently. Finally, 936 complete and valid questionnaires were returned (93.04% effective rate; mean age 15.61 ± 1.81 years). Of these, 462 were male, accounting for 49.4%, and 474 were female, accounting for 50.6%; 95 were only children, accounting for 10.1%, and 841 were non-only children, accounting for 89.9%; 174 were urban, accounting for 18.6%, and 762 were rural, accounting for 81.4%; 340 were freshman students, accounting for 36.3%, 352 were sophomores, accounting for 37.6%, and 244 in the senior year, accounting for 26.1%.

### 2.2. Materials

#### 2.2.1. Meaning in life

The level of meaning in life was assessed using the Meaning in Life Scale (Xinqiang, 2013). The scale consists of 10 items that include two dimensions: sense of having meaning and sense of seeking meaning. Among them, the sense of having meaning focuses on the extent to which individuals perceive the meaning of life, while the sense of seeking meaning focuses on the individual’s drive to actively pursue meaning. Participants answered each item on a seven-point Likert scale (1 = very unlikely to meet, 7 = very likely to meet). Higher scores indicate higher levels of meaning in an individual’s life. In this study, Cronbach’s α was 0.85, whereas those of the two dimensions constituting the scale were 0.80 and 0.87, respectively.

#### 2.2.2. Self-control

The Self-Control Scale was used to assess adolescent self-control (Wang Hongjiao, 2004). The questionnaire consists of three dimensions: emotional self-control, behavioral self-control, and thinking self-control, with a total of 36 items. Participants answered each item using a five-point Likert scale (1 = very inconsistent, 5 = very consistent) to calculate the total score. The higher the score on the questionnaire, the better the students’ self-control. The Cronbach’s α of the scale in this study was 0.88.

#### 2.2.3. Perceived social support

The Perceived Social Support Scale was used to measure perceived social support (Wang et al., 1999). The questionnaire consists of three dimensions: perceived family support, perceived friend support, and perceived support from others. The questionnaire is composed of 12 items, with four items per dimension. Participants answered each item using a seven-point Likert scale (1 = strongly disagree, 7 = strongly agree). Higher scores on the questionnaire indicate a greater ability to perceive social support. In this study, Cronbach’s α was 0.86, while those of the three dimensions constituting the scale were 0.80, 0.83, and 0.83, respectively.

### 2.3. Statistical processing and analysis plan

The questionnaire data were entered through EpiData 3.1, the data were collated and analyzed using SPSS 21.0, the mediating effect of navigating social support was analyzed using model 4 in the SPSS macro program PROCESS V3.5 developed by Hayes, and further analysis of whether gender plays a moderating role in the second half of the mediating effect of navigating social support was carried out using model 14.

## 3. Results

### 3.1. Common method bias test

Considering that the data in this study were collected from the subjects’ independent reports, the possibility of common method bias cannot be ruled out, although the administration procedure was strictly controlled (Zhou, 2004). Therefore, the validity of procedural control was examined using Harman’s one-way factorial analysis. All items of the predictor variables were put into an exploratory factor analysis, and the results yielded 14 factors with characteristic roots greater than 1, which explained 56.94% of the variance, and the explanation rate of the first factor was 16.42%, which was far below the threshold of 40%, indicating that the common method bias in this study was still within the acceptable range.

### 3.2. Descriptive statistics and correlation analysis

Descriptive statistical analysis showed that meaning in life was significantly and positively correlated with perceived social support (*r* = 0.36, *p* < 0.01) and self-control (*r* = 0.32, *p* < 0.01), but not with gender (*r* = 0.05, *p* > 0.05). Perceived social support was significantly and positively correlated with self-control (*r* = 0.25, *p* < 0.01) but not with gender (*r* = −0.04, *p* > 0.05). Self-control was significantly positively correlated with gender (*r* = 0.09, *p* < 0.01; see Table 1).

**Table 1:** Descriptive statistics and correlation analysis of the predictor variables.

	M ± SD	1	2	3	4
Gender	——	1			
Meaning in life	4.55 ± 0.91	0.05	1		
Perceived social support	4.81 ± 0.96	−0.04	0.36^**^	1	
Self-control	3.04 ± 0.50	0.09^**^	0.32^**^	0.25^**^	1

Gender is a virtual variable, male = 1, female = 2; ^**^P < 0.01.

### 3.3. Testing for the mediating effect of perceived social support

Based on the theoretical hypotheses, a model of the mediating effect of perceived social support between meaning in life and self-control was constructed. The mediating effects were analyzed using model4 in the PROCESS macro program developed by Hayes, and the results are shown in Table 2. Controlling for gender, meaning in life significantly and positively predicted perceived social support (*β* = 0.36, *t* = 11.63, *p* < 0.001). After including the mediating variable perceived social support, meaning in life still significantly and positively predicted self-control (*β* = 0.26, *t* = 7.92, *p* < 0.001), and perceived social support significantly and positively predicted self-control (*β* = 0.16, *t* = 4.99, *p* < 0.001; see Table 2).

**Table 2:** Analysis of the mediating role of perceived social support between meaning in life and self-control.

Regression equation		Fits the index			Significance of regression coefficient		
Outcome variable	Predictive variable	*R*	*R^2^*	*F*	*β*	*SE*	*t*
Perceived social support	Meaning in life	0.36	0.13	69.80^***^	0.36	0.03	11.73^***^
	Gender				−0.12	0.06	−2.00^*^
Self-control	Meaning in life	0.36	0.13	47.41^***^	0.26	0.03	7.92^***^
	Perceived social support				0.16	0.03	4.99^***^
	Gender				0.17	0.06	2.82^**^

^*^P < 0.05, ^**^P < 0.01, ^***^P < 0.001.

The bias-corrected Bootstrap method is considered to be an important method for testing the significance of the mediation effect, setting up to draw Bootstrap samples 5,000 times and observing the Bootstrap confidence intervals and standard errors of the parameter estimates, if they do not contain 0 in the 95% confidence interval, the results are said to be statistically significant and the mediation effect is significant. Therefore, this study first standardized each predictor variable, after which the significance of the mediation effect was tested using the bias correction Bootstrap method. The results are shown in Table 3, where none of the paths corresponded to a 95% confidence interval containing 0, and the total, direct, and indirect effects were statistically significant (all *p* < 0), indicating that perceived social support partially mediates the relationship between meaning in life and self-control. Further analysis revealed that the total effect of meaning in life on self-control was 0.32 (0.26 + 0.06) and the mediating effect of perceived social support was 18.75% (0.06/0.32*100%; see Table 3).

**Table 3:** Decomposition table of the mediating role of perceived social support.

Self-control	Normalized effect values	Bootstrap standard error	The 95% confidence interval		Relative effect value
			Superior limit	Lower limit	
Total effect	0.32	0.03	0.26	0.38	
Direct effect	0.26	0.03	0.20	0.32	81.25%
Mediating effect of perceived social support	0.06	0.01	0.03	0.08	18.75%

### 3.4. Testing for the moderating effect of gender

Model 14 in the SPSS macro program PROCESS, prepared by Hayes, was used to test the mediation model with moderation. It was explored whether gender plays a moderating role in the second half of the pathway mediated by perceived social support. The test results are listed in Table 4. Meaning in life was a significant predictor of self-control (*β* = 0.36, *t* = 11.63, *p* < 0.001). The predictive effect of perceived social support on self-control was not significant (*β* = −0.03, *t* = −0.31, *p* > 0.05). The effect of gender on self-control was significant (*β* = 0.17, *t* = 2.82, *p* < 0.05). The product term of perceived social support and gender had a significant positive predictive effect on self-control (*β* = 0.13, *t* = 2.12, *p* < 0.05). The 95% confidence interval did not include 0, indicating that the predictive effect of perceived social support on self-control was moderated by gender. The judgment indicator INDEX was 0.05, and the confidence interval was [0.002,0.091], and the confidence interval did not include 0, indicating that gender has a moderating effect on the predictive effect of meaning in life on self-control through the perceived social support. The effect of perceived social support on self-control was greater for females than for males (see Table 4).

**Table 4:** Regression analysis of the moderating effect of gender.

Regression equation		The overall fit index			Significance of regression coefficient				
Outcome variable	Predictive variable	*R*	*R^2^*	*F*	*β*	*SE*	CI lower limit	CI superior limit	t
Perceived social support	Meaning in life	0.36	0.13	135.17^***^	0.36	0.03	0.30	0.42	11.63^***^
Self-control	Meaning in life	0.37	0.14	36.81^***^	0.25	0.03	0.19	0.32	7.91^***^
	Perceived social support				−0.03	0.10	−0.22	0.16	−0.31
	Gender				0.17	0.06	0.05	0.29	2.82^*^
	Perceived social support ^*^ gender				0.13	0.06	0.01	0.25	2.12^*^

Each variable in the model adopts standardized variables, the same below. ^*^P < 0.05, ^***^P < 0.001.

**Figure 2:**
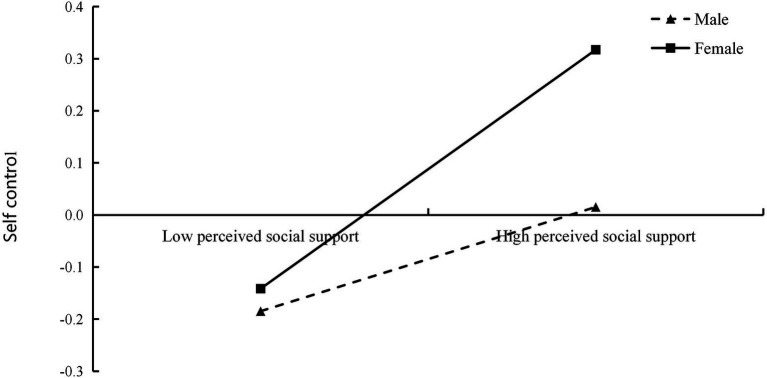
The moderating role of gender in perceived social support and self-control.

To understand the essence of the interaction effect between perceived social support and gender, male and female subjects were analyzed separately, and the mediated effect values and 95% Bootstrap confidence intervals of perceived social support between meaning in life and self-control for both groups are shown in Table 5. The moderating role of gender between perceived social support and self-control was further analyzed using the simple slope method (Fang et al., 2015). The results are shown in Figure 2. Perceived social support in females had a significant positive predictive effect on self-control (simple slope = 0.23, *t* = 5.08, *p* < 0.001). Perceived social support in males still had a significant positive predictive effect on self-control (simple slope = 0.10, *t* = 2.26, *p* < 0.05).

**Table 5:** The mediation effects of social support.

Metavariable	Gender	Effect value	Boot standard error	BootCI superior limit	BootCI lower limit
Perceived social support	Male	0.04	0.02	0.003	0.073
	Female	0.08	0.02	0.050	0.118

**Figure 3:**
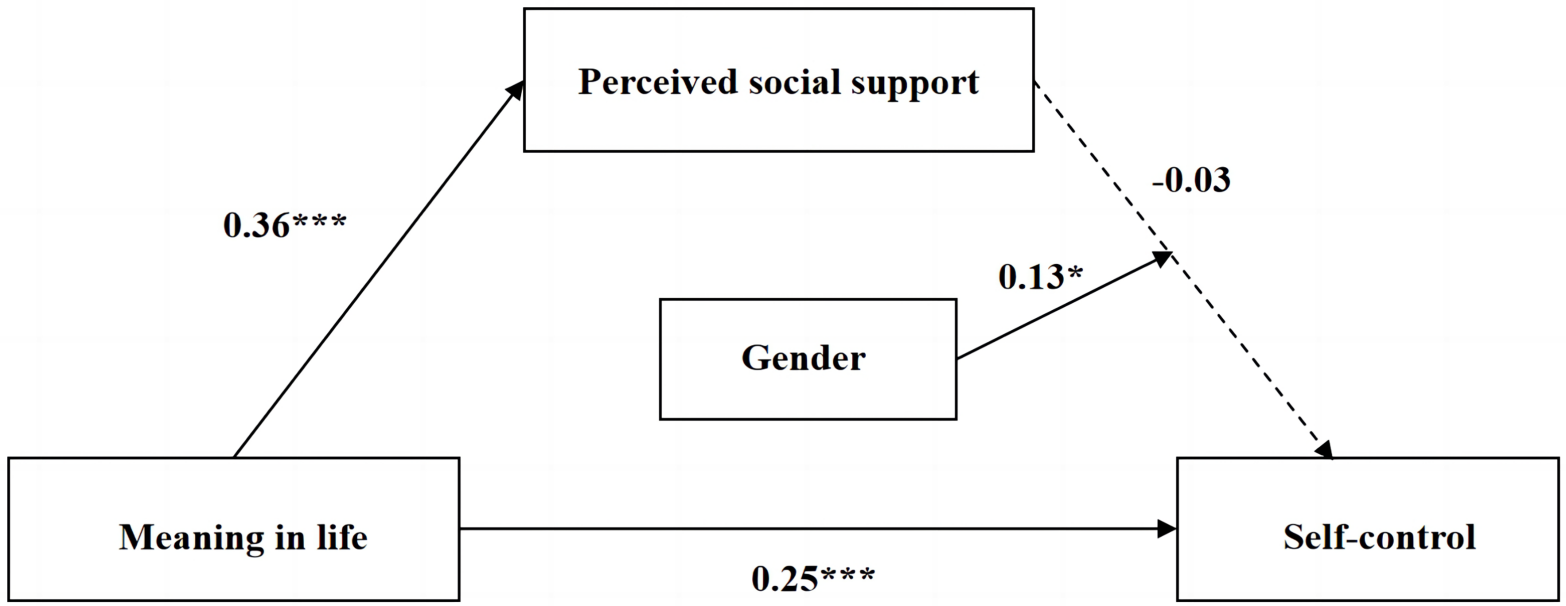
A moderated mediation model of the gender. ^*^The correlation is significant at the 0.05 level. ^***^The correlation is significant at the 0.01 level.

In summary, the moderated mediation model proposed in this study was empirically supported. Perceived social support mediated the relationship between meaning in life and self-control, and the second half of this mediating role was moderated by gender factors. The adjusted model relationships are shown in Figure 3.

## 4. Discussion

### 4.1. The relationship between meaning in life and self-control

The current findings support Hypothesis 1, which suggests meaning in life is positively related to adolescent self-control and perceived social support. The results of this study suggest that meaning in life significantly and positively predicts adolescent self-control, which is consistent with the existing research (Shao, 2021). Frankl’s theory of meaning in life suggests that meaning in life plays a positive role by providing individuals with value, direction, and control over events in their lives (Frankl and He Zhongqiang, 2003). The stronger the meaning in life, the more individuals are able to perceived the value of their own existence and have greater self-control to regulate undesirable emotions and behaviors (Zhang and Zhang, 2021). Emotional attribution theory can also explain this finding. This theory suggests that for physiological arousal to be converted into an emotion, there must be not only feedback from the physiological response, but also a marker of the situation that leads to the physiological response (Schachter and Singer, 1962). Specifically, when individuals mark themselves as beings with a robust meaning in life, they can access extremely rich inner kinetic energy and subsequently invoke the inner purpose system to regulate their external behavior and performance, suppress instinctive impulsive desires, and strengthen inner self-control. At the same time, individuals who tend to seek meaning in life place more emphasis on achieving long-term goals and thus exhibit a more pronounced delayed gratification mentality. In this process, they shift their attention from external things to internal changes, social and situational aspects of the performance situation. At this time, individuals have stronger self-focused attention, more emphasis on self-care and self-evaluation, more motivation to adjust their behavior, and are more likely to achieve their life goals (Wicklund and Duval, 1971), and have greater self-control.

### 4.2. Mediating role of perceived social support

Consistent with Hypothesis 2, we found that perceived social support is a mediator in the relationship between meaning in life and adolescent self-control. The present study also found that perceived social support could serve as a bridge to realize the impact of meaning in life on adolescent self-control. The main source of the mediating role of perceived social support may be that, on the one hand, experiencing more social support can enhance adolescents’ self-control. It has been suggested that the imbalance between psychological and physical development makes adolescents irrational, impulsive, and emotionally vulnerable, and therefore adolescents are more sensitive to external social support. Simultaneously, more social support means being accepted and recognized by others, which can lead to a better self-image. The maintenance of the image, in turn, motivates adolescents to show higher self-control when dealing with problems and to be more willing to behave in accordance with social rules (Yang, 2021). In addition, in complex relationships with family and friends, individuals acquire the message of being loved and cared for by social support, which promotes the development of emotion management and executive functioning, thereby effectively improving self-control (Feng Kong and You, 2013). On the other hand, meaning in life influences adolescents’ ability to perceive social support. It has been found that strong meaning in life predicts a more positive level of psychological well-being, and individuals with higher levels of psychological well-being have superior interpersonal skills, which motivates them to maintain close and friendly interactions with their peers (Kang et al., 2020), easily perceiving goodwill in interpersonal interactions, and thus are able to experience and receive more social support. At the same time, a strong meaning in life also indicates that individuals have more hope for the future, so individuals will maintain a positive mindset to interact with others, avoiding interpersonal conflicts and establishing stable relationships (Chen Yidi, 2018). This may reveal that the perception of social support and the meaning in life is extremely important for the development of adolescents’ self-control. By strengthening life-meaning education and improving individuals’ perceptions of social support can effectively stimulate adolescents’ self-control ability.

### 4.3. Moderating role of gender

The results indicate that gender differences play a moderating role in the second half of the pathway through which meaning in life affects adolescents’ self-control through perceived social support, which is consistent with Hypothesis 3. We found gender differences in the effect of perceived social support on adolescent self-control, with females’ perceived social support having a more significant effect on their self-control than males’. The reason for this is, on the one hand, the difference in psychological development between the two groups. Specifically, during adolescence, females are more delicate and more likely to perceive support around them than males, and thus their psychological development is more mature, assertive, and capable of self-control (Jiandan, 2012; Jingmin, 2012). On the other hand, it lies in the different gender role expectations shaped by the culture of the society. It is noteworthy that such social expectations create differential social evaluations and have a more pronounced impact on female. Female’s greater reliance on interpersonal relationships and the social environment makes them more willing to conform their behavior to social expectations, and for this reason they will continue to strengthen their self-control. Men, in contrast, are more focused on their own development, tend to rely on self-imposed norms, and are less influenced by social evaluations (Rose and Karen, 2006), thus leading to a reduced need for continuous improvement of self-control and a weakening of self-control.

Therefore, based on gender differences in the influence of perceived social support on adolescents’ self-control, we need to follow the rules of psychological development of both genders, adopt scientific teaching methods, and provide targeted knowledge to promote the development of strengths and weaknesses of each gender group. At the same time, in education, we should pay more attention to females’ emotional demands and focus on cultivating males’ rational consciousness and awareness of rules, so as to improve the psychological quality of self-control and emotional regulation of adolescents and thus promoting the transformation of students from “other discipline” to “self-control.”

### 4.4. Limitations and implications

This study reveals that perceived social support mediates the relationship between meaning in life and self-control along with gender differences, further revealing the mechanisms underlying the influence of meaning in life on adolescents’ self-control. The results of this study provide an important empirical basis for nurturing adolescents’ sense of self-control from the perspective of the meaning in life. In order to enhance adolescent self-control, we should not only strengthen adolescents’ life-meaning education and reinforce their expectation of life meaning, but also elevate adolescents’ ability to perceive social support so as to obtain rich social support around them. At the same time, gender differences should be taken into account when nurturing adolescents’ self-control abilities. We need to tailor the developmental program to the gender-specific adolescents in order to fully utilize the cultivating effect.

In addition, the present findings also suggest some limitations and several directions for future research. Firstly, meaning in life, self-control, and perceived social support are all multidimensional variables, and the analysis based on the dominant variables in this study can hardly reveal the more complex relationships among them. Future studies can use these three as latent variables and construct more complex latent variable interaction effect models through standardized estimation of latent variables to explain the interaction effects between variables in more detail as well as to compare simple main effects. Secondly, the results of this study originated from cross-sectional studies, which are difficult to reveal the causal relationships between variables or the dynamic patterns of self-control development in adolescent groups. Future follow-up studies can be used to further analyze the effects of meaning in life and perceived social support on the development of adolescent self-control and to construct a dynamic model of growth and development. Finally, development is a continuous dynamic process, and environmental factors also play a role. As both meaning in life and perceived social support belong to personal factors, future investigations can combine personal and environmental factors to explore the mechanism of self-control in more depth and provide rich theoretical support for developing intervention programs to enhance adolescents’ self-control.

## 5. Conclusion

The findings of this study are as follows. The present study found that meaning in life significantly and positively predicted self-control and perceived social support. Perceived social support partially mediated the relationship between meaning in life and self-control. Gender difference moderated the second half of the pathway of the mediation model of meaning in life on self-control, as evidenced by the fact that perceived social support was a stronger predictor of self-control for females than for males. The results of this study implied that enhancing perceived social support could help adolescents with lower meaning in life to enhance self-control, and this process was more significant in the group of girls.

## Data availability statement

The raw data supporting the conclusions of this article will be made available by the authors, without undue reservation.

## Ethics statement

The studies involving human participants were reviewed and approved by the First Affiliated Hospital of Shihezi University, Shihezi University. Written informed consent to participate in this study was provided by the participants’ legal guardian/next of kin.

## Author contributions

YL and SD designed the experiment, collected data, prepared the manuscript, and made data analysis. YS corrected the whole language of the manuscript and made final approval. CM gave technique supports and valuable suggestions in experiment designing. All authors contributed to the article and approved the submitted version.

## Funding

This research was supported by the Shihezi University Graduate Education Teaching Reform Research Project of China (2021Y-JGSJ11).

## Conflict of interest

The authors declare that the research was conducted in the absence of any commercial or financial relationships that could be construed as a potential conflict of interest.

## Publisher’s note

All claims expressed in this article are solely those of the authors and do not necessarily represent those of their affiliated organizations, or those of the publisher, the editors and the reviewers. Any product that may be evaluated in this article, or claim that may be made by its manufacturer, is not guaranteed or endorsed by the publisher.

## References

[ref1] Barbara R. S. Pierce G. R. Edward N. S. Sarason I. J. Waltz J. A. Poppe L. (1991). Perceived social support and working models of self and actual others. J. Pers. Soc. Psychol. 60, 273–287.

[ref2] Bernadette V. Tatjana S. (2019). Bringing giftedness to bear: generativity, meaningfulness, and self-control as resources for a happy life among gifted adults. Front. Psychol. 10:1972. doi: 10.3389/fpsyg.2019.01972, PMID: 31572251 PMC6753398

[ref3] Brassai L. Piko B. F. Steger M. F. (2011). Meaning in life: is it a protective factor for adolescents’ psychological health? Int. J. Behav. Med. 18, 44–51. doi: 10.1007/s12529-010-9089-6, PMID: 20960241

[ref4] Chen Yidi G. Y. (2018). Harmonious personality, social support, self-control and work-family conflict - a comparison of 3 models based on occupational groups. J. Peking Univ. 54, 1123–1132. doi: 10.13209/j.0479-8023.2018.049

[ref5] Ding Qian Z. Y. Zongkui Z. (2020). Relative deprivation and college students’ online excesses:the mediating role of self-loss and gender differences. Psychol. Dev. Educ. 36, 200–207. doi: 10.16187/j.cnki.issn1001-4918.2020.02.09

[ref6] Fang J. Wen Z. Liang D. Li N. N. (2015). Analysis of moderating effects based on multiple regression. Psychol. Sci. 38, 715–720.

[ref7] Feng Kong J. Z. You X. (2013). Self-esteem as mediator and moderator of the relationship between social support and subjective well-being among Chinese university students. Soc. Indic. Res. 112, 151–161. doi: 10.1007/s11205-012-0044-6

[ref8] Fitzsimons G. M. Finkel E. J. (2011). “The effects of self-regulation on social relationships,” in Handbook of self-regulation: Research, theory, and applications. eds. Vohs K. D. Baumeister R. F. (New York, NY: Guilford Press), 407–421.

[ref9] Frankl V. He Zhongqiang Y. F. (2003). In pursuit of the meaning of life. Beijing: Xinhua Publishing House.

[ref10] Hamama L. Hamama-Raz Y. (2021). Meaning in life, self-control, positive and negative affect: exploring gender differences among adolescents. Youth Soc. 53, 699–722. doi: 10.1177/0044118X19883736

[ref11] Hanxiao X. (2008). The effect of navigating social support on college students’ depression. Chin. J. Health Psychol. 4, 415–417. doi: 10.13342/j.cnki.cjhp.2008.04.047

[ref12] Hedlund D. E. (1977). Personal meaning: the problem of educating for wisdom. Personnel Guid. J. 55, 602–604. doi: 10.1002/j.2164-4918.1977.tb04312.x, PMID: 34564295

[ref13] Hu Qian T. T. Wenbin G. Fan C. Wang L. Cao J. Cai Y.. (2022). A systematic review of adolescent self-control research. Chin. J. Mental Health 36, 129–134.

[ref14] HY Jung K. L. (2008). Structural equation modeling of perceived social support, self-control and subjective well-being of children. Korean J. Child Stud. 29, 167–179.

[ref15] Jiandan S. (2012). A study on the relationship between social support and well-being of students in higher education Suzhou university. Suzhou: Suzhou University Press.

[ref16] Jia L. (2016). Meaning of life education for adolescents in the perspective of social control. Psychol. Explor. New 36, 392–396.

[ref17] Jingmin Z. (2012). A study on the relationship between self-esteem, perceived social support and interpersonal trust among college students. Hebei Normal University.

[ref18] Jong Jae Kim S. W. K. (2012). The relation between the meaning of the life and game addiction: the mediated effects of self-efficacy and self-control. Korean J. Youth Stud. 19:12.

[ref19] Kang S. J. Ko S. H. Kim J. Y. Kim S. R. (2020). Effects of a mental fitness positive psychology intervention program on inpatients with schizophrenia in South Korea: a feasibility study. Perspect. Psychiatr. Care 56, 6–13. doi: 10.1111/ppc.12332, PMID: 30430580

[ref20] Kochanska G. Coy K. C. Murray K. T. (2001). The development of self-regulation in the first four years of life. Child Dev. 72, 1091–1111. doi: 10.1111/1467-8624.00336, PMID: 11480936

[ref22] Nakash O. Arnon S. Hayat T. Abu Kaf S. (2022). Strength of social ties and perceived tangible support: distinct characteristics and gender differences of older adults’ social circles. J. Women Aging 34, 719–730. doi: 10.1080/08952841.2021.1951075, PMID: 34255616

[ref23] Nie Hanying G. Y. (2017). The relationship between self-concept clarity and sense of meaningfulness of life and subjective well-being. Chin. J. Clin. Psych. 25, 923–927. doi: 10.16128/j.cnki.1005-3611.2017.05.029

[ref24] Nofziger S. Callanan V. J. (2016). Predicting suicidal tendencies among high risk youth with the general theory of crime. Deviant Behav. 37, 167–183. doi: 10.1080/01639625.2014.1004029

[ref25] Oliva A. Antolin-Suarez L. Rodriguez-Meirinhos A. (2019). Uncovering the link between self-control, age, and psychological maladjustment among spanish adolescents and young adults. Psychosoc. Interv. 28, 49–55. doi: 10.5093/pi2019a1

[ref26] Orkibi H. Hamama L. Gavriel-Fried B. Ronen T. (2018). Pathways to adolescents’ flourishing: linking self-control skills and positivity ratio through social support. Youth Soc. 50, 3–25. doi: 10.1177/0044118X15581171

[ref27] Qiu Yiwen L. Y. Yi L. (2021). Adolescent depression: a social support-based perspective. Psychol. Dev. Educ. 37, 288–297. doi: 10.16187/j.cnki.issn1001-4918.2021.02.16

[ref28] Rose A. J. R. Karen D. (2006). A review of sex differences in peer relationship processes: potential trade-offs for the emotional and behavioral development of girls and boys. Psychol. Bull. 132, 98–131. doi: 10.1037/0033-2909.132.1.98, PMID: 16435959 PMC3160171

[ref29] Schachter S. Singer J. E. (1962). Cognitive, social, and physiological determinants of emotional state. Psychol. Rev. 69, 379–399. doi: 10.1037/h0046234, PMID: 14497895

[ref30] Schnell T. Krampe H. (2020). Meaning in life and self-control buffer stress in times of COVID-19: moderating and mediating effects with regard to mental distress. Front. Psych. 11:582352. doi: 10.3389/fpsyt.2020.582352, PMID: 33173525 PMC7538834

[ref31] Shao W. Q. (2021). Adolescent fathers’ emotional warmth and sense of meaning in life: multiple mediators of self-control and emotion regulation. Shanhaijing Front. Educ. 32, 0250–0251.

[ref32] Stillman T. F. Baumeister R. F. Lambert N. M. Crescioni A. W. DeWall C. N. Fincham F. D. (2009). Alone and without purpose: life loses meaning following social exclusion. J. Exp. Soc. Psychol. 45, 686–694. doi: 10.1016/j.jesp.2009.03.007, PMID: 20161218 PMC2717555

[ref33] Sun F. K. Wu M. K. Yao Y. Chiang C. Y. Lu C. Y.. (2021). Meaning in life as a mediator of the associations among depression, hopelessness and suicidal ideation: a path analysis. J. Psychiatr. Ment. Health Nurs. 29, 57–66. doi: 10.1111/jpm.12739 33559221

[ref34] Ting Z. (2021). The influence of junior high school students’ sense of meaning in life on time management tendencies: The mediating role of self-control [dissertation]. [Yangzhou (China)]: Yangzhou University.

[ref35] Touré-Tillery M. Fishbach A. (2015). It was(n’t) me: exercising restraint when choices appear self-diagnostic. J. Pers. Soc. Psychol. 109, 1117–1131. doi: 10.1037/a0039536, PMID: 26414839

[ref36] Van den Akker A. L. Dekovic M. Asscher J. J. Shiner R. L. Prinzie P. (2013). Personality types in childhood: relations to latent trajectory classes of problem behavior and overreactive parenting across the transition into adolescence. J. Pers. Soc. Psychol. 104, 750–764. doi: 10.1037/a0031184, PMID: 23276273

[ref38] Wang X. Wang X. Ma H. (1999). Handbook of mental health rating scales. Beijing: China Mental Health, 127–133. doi: 10.16719/j.cnki.1671-6981.2004.06.055

[ref37] Wang Hongjiao L. J. (2004). Development of a self-control questionnaire for secondary school students and its investigation. Psychol. Sci. 6, 1477–1482.

[ref21] Wenzel M. Bürgler S. Rowland Z. Hennecke M. (2021). Self-control dynamics in daily life: the importance of variability between self-regulatory strategies and strategy differentiation. Eur. J. Pers 08902070211043023

[ref39] Wicklund R. A. Duval S. (1971). Opinion change and performance facilitation as a result of objective self-awareness. J. Exp. Soc. Psychol. 7, 319–342. doi: 10.1016/0022-1031(71)90032-1

[ref40] Xinqiang W. (2013). Reliability and validity of the revised Chinese version of the sense of life meaning scale in a population of secondary school students. Chin. J. Clin. Psych. 21, 764–767. doi: 10.16842/j.cnki.issn2095-5588.2016.04.003

[ref41] Yang S.-L. (2021). The relationship between social support and problem behavior in high school students: the mediating role of self-control. J. Gannan Normal Univ. 42 1–6. doi: 10.13698/j.cnki.cn36-1346/c.2021.05.017

[ref42] Ye Baojuan H. X. Qiang Y. Zhujing H. (2014). Mechanisms of comprehending the effects of social support, coping efficacy, and stressful life events on adolescents’ academic achievement. Psychol. Sci. 37, 342–348. doi: 10.16719/j.cnki.1671-6981.2014.02.017

[ref43] Yek M. H. Olendzki N. Kekecs Z. Patterson V. Elkins G. (2017). Presence of meaning in life and search for meaning in life and relationship to health anxiety. Psychol. Rep. 120, 383–390. doi: 10.1177/0033294117697084, PMID: 28558607

[ref44] Youngoh Jo L. B. (2014). Stability of self-control and gender. J Crim Just 42, 356–365. doi: 10.1016/j.jcrimjus.2014.05.001

[ref47] Zhang C. Wang C. Zhai L. (2016). The effect of college students’ self-control ability on academic procrastination: with moderating mediating effects. Psychol. Techniq. Appl. 4, 209–214. doi: 10.16842/j.cnki.issn2095-5588.2016.04.003

[ref49] Zhang V.-W. Zhang Y.-Z. (2021). Perceived social support and depression among college students: the role ofmeaning in life and self-control. J. Inner Mongolia Normal Univ. 50, 82–87.

[ref45] Zhang Shupeng Z. Q. Caina L. (2015). A meta-analysis of gender differences in navigating social support. Psychol. Dev. Educ. 31, 393–401. doi: 10.16187/j.cnki.issn1001-4918.2015.04.02

[ref46] Zhang Shuyue X. Y. Haoqiang Y. (2010). The connotation, measurement and function of life meaning. Dvances. Psychol. Sci. 18, 1756–1761.

[ref48] Zhang Xiuge Q. J. Wenyu H. (2019). The relationship between college students’ sense of meaning in life and their tendency to mobile phone addiction: the mediating role of self-control. Psychol. Behav. Res. 17, 536–545.

[ref50] Zhou H. (2004). Statistical tests and control methods for common method bias. Adv. Psychol. Sci. 6, 942–950.

[ref51] ZN L. (2019). The influence of older people’s sense of meaning in life on attitudes toward death: The mediating role of social support. dissertation. Jilin (China): Jilin University.

